# Anti-reflectance investigation of a micro-nano hybrid structure fabricated by dry/wet etching methods

**DOI:** 10.1038/s41598-018-26234-6

**Published:** 2018-05-18

**Authors:** Xiao Tan, Zhi Tao, Mingxing Yu, Hanxiao Wu, Haiwang Li

**Affiliations:** 1 School of Energy and Power Engineering, Beihang University, Beijing, 100191 China; 2 National Key Laboratory of Science and Technology on Aero Engine Aero-thermodynamics, Beijing, 100191 China; 3 The Collaborative Innovation Center for Advanced Aero-Engines of China, Beijing, 100191 China

## Abstract

Black silicon fabrication and manipulation have been well reported by institutes around the world and are quite useful for solar absorption and photovoltaic conversion. In this study, silicon micro-nano hybrid structures were fabricated, and the morphologies of the hybrid structures were analyzed. This paper studied nanostructures formed on tips, pits and a flat surface using a dry etching method and a wet etching method. In terms of nanostructure morphology, nanostructures etched by the wet etching method (13 μm) were taller than those etched by the dry etching method (1 μm), but the wet etched morphology was less organized. After the nanostructures were grown, six samples with nano sturctures and three samples with micro sturctures were measured by a photometer for reflectivity testing. The nine samples were compared and analyzed using the integral of reflectivity and solar emissivity at the earth’s surface. The results show that the nanostructures grown on a tip surface using the wet etching method had the minimum reflectivity in the wavelength range of 300 nm–1100 nm, in consideration of the forbidden energy gap of silicon.

## Introduction

Solar cells are essential in applications of energy harvesting and energy storage. To enhance the efficiency of a solar cell, low surface reflectance of solar cells is required to maximize the number of incident photons absorbed by the semiconductor that converts light to electrical energy. In recent years, many studies on methods for reducing the surface reflectance of a solar cell have been reported, including the coating method, dry etching method, and wet etching method. The coating method is a standard technique that reduces the reflectance in crystalline Si solar cells and other solar cells by using a quarter-wavelength antireflection (AR) coating on the top surface^1–3^. However, the coating method results in destructive performance on the surface and inhibits the photon conversion process, thereby reducing the efficiency of the solar cell. The dry etching method for fabricating black silicon uses SF_6_-O_2_ plasma and was proposed in 1995 as a tool to identify optimal conditions for vertical silicon deep etching^4^. In 2001, Zaidi *et al*. studied a solar cell textured by reactive ion etching (RIE), but paid little attention to microstructures^5^. Temperature is an important parameter that influences the growth conditions in dry etching. While black silicon can generally be fabricated at low (−40 to −30 °C)^6^ or even cryogenic temperatures^7^, Pezoldt *et al*. presented nanostructure fabrication at temperatures between 20 and 30 °C^8^. However, fabrication at temperatures between −30 and 20 °C, i.e., the operating temperature for a normal RIE machine or ICP (inductive coupled plasma), is rarely reported. The wet etching method typically uses Ag or Au for nanoparticle catalysis and HF for etching substances^9–13^. This method is a simple and quick approach for producing a large amount of nanostructure grass layers. However, the wet etching method may cause large area defects and is less stable than the dry etching method. Hsu C. H. *et al*. investigated fabrication and characteristic of black silicon for solar cell applications before 2014 and wrote an overview of them^14^. At the same time, in 2014, Liu X. reviewed properties of solar energy applications using black silicon^15^. Both of them recognized the black silicon as an important technique for energy conservation and collection and thought it would be a promising technology. In addition to optical application^16–18^, other fields, including Biology^19^, heat transfer^20^, photoelectrochemical^21^, electroosmotic flow in micro channel^22^ etc., also take the full advantage of black silicon.

Bernhard discovered that the corneas of moth eyes have a nanostructured surface that acts as an antireflective medium, realizing night camouflage^23^. Since then, this so-called ‘moth eye effect’ has been widely studied and reported^24–30^. To understand the underlying physical principles, the optical properties of nanostructured surfaces have been investigated^31–38^. Surface roughening for anti-reflective function consists of numerous fabrication methods, including coating method (sol-gel processing^39–43^, dip^44^ and spin coating^45^ etc.), glancing angle deposition (GLAD)^46–49^, Chemical vapor deposition (CVD)^50–53^, etching method (wet^54,55^ and dry method^56,57^), and lithography method^58–60^ etc. Silicon-base surface usually uses etching method, photolithography method and femtosecond (fs) laser method. However, photolithography method requires special mask to accomplish patterns and femtosecond laser method needs high density energy to fabricate patterns. Etching method, including wet and dry etching method, is convenient to fabricate micro and nano structures on silicon wafer, and hence it becomes popular in manufacturing rough surface for anti-reflectance use. In recent articles, rough surface on silicon using etching method indicated excellent property of anti-reflectance^13,61–64^. Particularly, Anti-reflective surface was applied to other field and was developed to be widely used in recent years. Saifeng Z. *et al*. reported a femtosecond (fs) laser microstructured silicon with Au film with using replication technique, which firstly showed great enhancement of infrared light absorption over a broad wavelength band (2.7~15.1 μm)^65^. In the reference, the highest infrared light absorption reached to 90% and infrared light absorption remained more than 60%. Zhang W. *et al*. studied a narrow-band reflectance filter using glancing-angle deposition technique and photolithography. In the study, the absorption of the filter was high only in a narrow wavelength range (620~630 nm) but was very low in other wavelength range (500~800 nm)^66^. Tan G. *et al*. demonstrated a broadband moth-eye-like AR surface on a flexible substrate, intended for flexible display applications. The motheye-like nanostructure was fabricated by an imprinting process onto a flexible substrate with a thin hard-coating film, which exhibited excellent AR with luminous reflectance <0.23% and haze below 1% with indistinguishable image quality deterioration^67^. Although various methods for nanostructure fabrication have been investigated in detail over the past decades, few articles have paid attention to micro-nano hybrid structures^68^, with nanostructures on the bottom of micro trenches and no grass in the profiles. Therefore, this paper focuses on the fabrication of micro-nano hybrid structures and their reflectance values. In addition, micro-nano hybrid structures are beneficial for absorbing the infrared spectrum of solar radiation, from which a solar cell can absorb more thermal energy. Such absorption may also reduce the forbidden gap of silicon because a negative correlation exists between temperature and the forbidden gap value. In this paper, using hybrid structures showed better performance (lower reflectance) than single structure using the same etching method^16,21^. The results of using hybrid structures indicated that reflectance kept low and stable among 300 nm~1000 nm but that of using single structure in other may showed that reflectance increased in small wavelength^2,69–71^.

Reflectance for normal incidence was measured on a UV3600 spectrophotometer. The morphology and structures of the samples were characterized with a scanning electron microscope (SEM), from SEC and Zeiss Company.

## Fabrication

**Figure 1:**
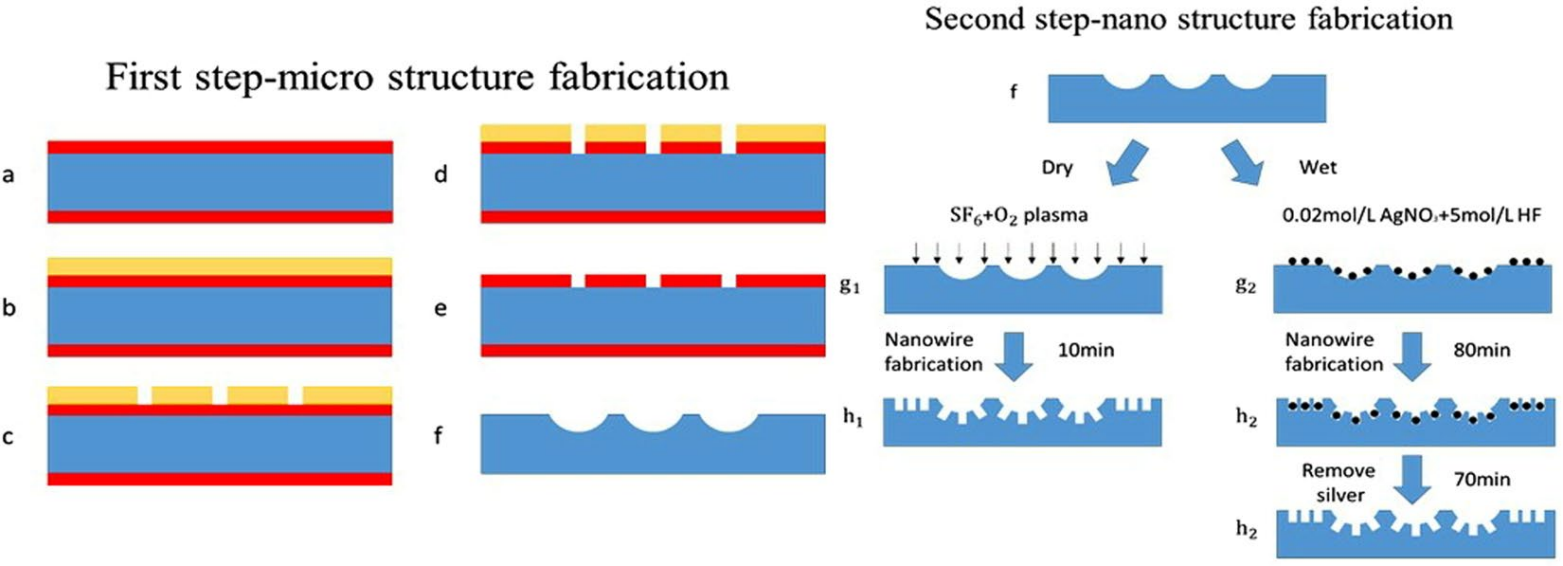
Process flow of hybrid structures. The hybrid structures include two steps. For the samples in paper, the first step is dry etching by ICP, fabricating the micro structures. In the second step, the samples could be treated by dry etching or wet etching, which shows different results of reflectance. For the details of recipe number, Table 1 indicates specific process flow of each number of sample.

This paper was designed to fabricate a kind of surface with high light-abortion efficiency. Nine samples were fabricated and process of them were different as shown in Table 1 and Fig. 1 below. These hybrid structures actually are secondary structures and they are intended to absorb more light rather than wafers with single micro or nano structures. Table 1 shows the group number and process details. The name of each sample in Table 1 is named according to the second step process method and micro structure shape.

**Table 1:** Process flow of nine samples.

Number/name	First step - micro structure fabrication	Second step - nano structure fabrication
1/wet-tip	Dry etching by ICP	Metal-assisted wet chemical etching
2/wet-pit	Dry etching by ICP	Metal-assisted wet chemical etching
3/wet-flat	No treatment	Metal-assisted wet chemical etching
4/dry-tip	Dry etching by ICP	Dry etching by ICP
5/dry-pit	Dry etching by ICP	Dry etching by ICP
6/dry-flat	No treatment	Dry etching by ICP
7/tips	Dry etching by ICP	No treatment
8/pits	Dry etching by ICP	No treatment
9/silicon	No treatment	No treatment

In this experiments, 4-inch silicon wafers with crystal orientation of 100, doping type of N, silicon wafer thickness of 500 μm, and silicon oxide thickness of 1 μm were used. As shown in Fig. 1, the first step was to fabricate micro structure, including micro tips and pits. The details of first step process flow are as below: (a) piranha (H_2_SO_4_/98%:H_2_O_2_/30% = 3:1) washing for 10 min, (b) spin coat photoresist, SPR220, in 3000 rpm and prebake 30 min in nitrogen atmosphere oven, (c) pattern photoresist with 10 mJ/cm^2^ ultraviolet light and post bake 15 min in nitrogen atmosphere oven, (d) Use buffer oxide etching fluid (HF:NH_4_F = 1:5) to etch silicon oxide without destruction of photoresist, (e) piranha (H_2_SO_4_/98%:H_2_O_2_/30% = 3:1) washing for 10 min to remove the photoresist, (f) finally, silicon wafers with micro tips or pits were fabricated. Samples were fabricated using two steps, with the aim of growing nanostructures on micro structures. The first step used a high-energy, high-density plasma for isotropic etching with SF_6_/O_2_ (130 sccm/13 sccm) to manufacture micro structures, tips and pits. In the second step, which applied two methods, nanostructures were fabricated on the tips and pits, as well as on a flat sample for comparison. Four types of micro-nano hybrid samples and two flat samples were fabricated, with three samples produced using the wet etching method and the other three samples obtained using the gas etching method in second step. Each method was performed to obtain microstructures with tips, pits and a flat surface. The wet etching method process in second step is as follows: (1) piranha (H_2_SO_4_/98%:H_2_O_2_/30% = 3:1) washing for 10 min, (2) solution (AgNO_3_/0.02-mol/L:HF/5-mol/L) configuring and soaking for 80 min, and (3) solution (HNO_3_/65%) configuring and soaking for 70 min. The dry etching method parameters are as follows: (1) gas flow: SF_6_/60 sccm:O_2_/80 sccm, (2) RF (radio frequency) power: 200 W, (3) bias power: 25 W, (4) temperature: 5 degrees centigrade, and (5) pressure: 10 mTorr.

**Figure 2:**
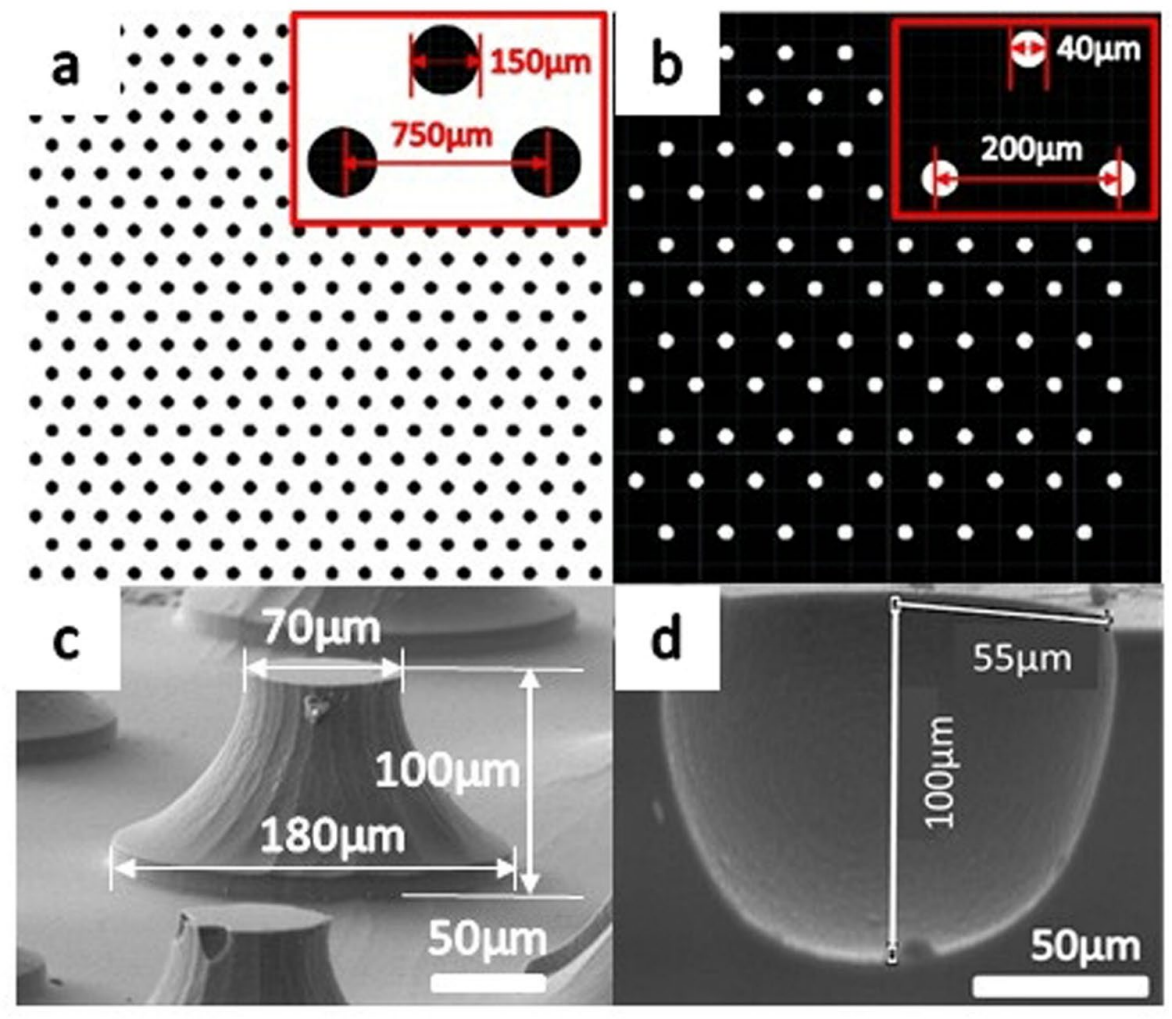
Mask patterns in first step process and fabrication results observed in SEM (**a**) the mask patterns of tips, (**b**) the mask patterns of pits, (**c**) fabrication results of tips observed in SEM, and (**d**) fabrication results of pits observed in SEM. Two kinds of three-dimension micro structures are patterned by mask layer shown in (**a**) and (**b**). The sizes of mask layer and micro structures are shown in (**a**)–(**d**). The two kinds of micro structures obtain the same curve profile by controlling the recipes of them. They were etched to 100 micrometer in depth and 55 micrometer in lateral side. Though they have the same curve profile, they indicated different reflective feature when grown with nano structures.

The tips were etched under the protection of mask shown in Fig. 2(a), pits were etched under the protection of mask shown in Fig. 2(b). In the Fig. 2(a,b), black parts represent mask patterns and white ones represent no mask patterns. Three center point of circles form an equilateral triangle and any distance between two center point is the same. After first step, micro three-dimension can be observed by SEM as shown in 2(c) and 2(d). It is found that the lateral etching rate and vertical etching rate determinate the curve profile of structures. The curve profile of tips was the same as that of pits through controlling the depth and lateral etching distance, as shown in Fig. 2(c,d).

**Figure 3:**
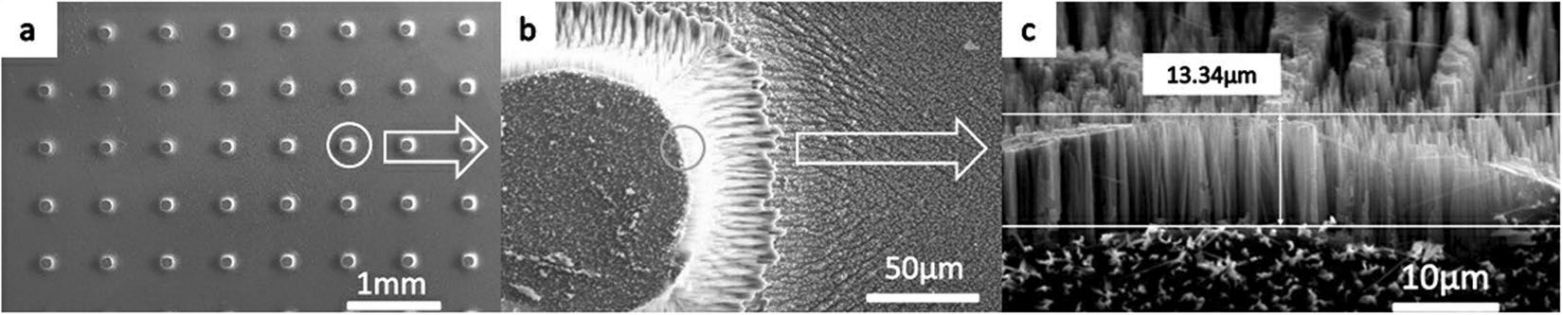
Nanostructures on micro tips obtained using the wet etching method: (**a**) top view of a low-magnification micrograph, (**b**) nanostructures on the top surface of the tips and the bottom surface of the substrate, and (**c**) nanostructures with a height of 13.34 μm on the profile of the tips. The tip sample with nanostructures that were fabricated by wet etching was indicated in Fig. 3, from millimeter scale to micrometer scale. High density nanograsses with height of 13.34 micron were seen in (**c**) and this sample showed the best performance of absorption among nine samples.

As shown in Fig. 3(a,b), nanostructure tips can be fabricated on the top surface of the tips, the profile of the tips and the bottom of the substrate. In addition, after 80 min of etching, the nanostructures grew 13.34 μm on the profile of the tips, as shown as Fig. 3(c). During fabrication, a few bubbles were observed and could not be removed by sonication. Even with a few seconds of sonication during fabrication, the solution of AgNO_3_ and HF would become turbid, and the HF could no longer etch silicon because the Ag cannot return to its original location.

**Figure 4:**
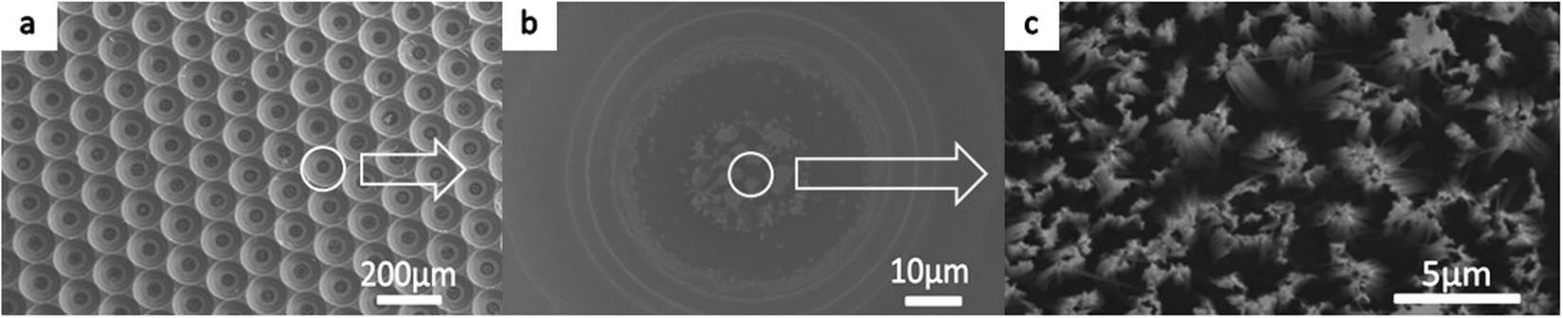
Nanostructures on micro pits obtained using the wet etching method: (**a**) top view low-magnification micrograph, (**b**) silver on the bottom surface of the pits, and (**c**) high-magnification SEM image of nanostructures on the top surface of the pits. The pit sample with nanostructures that were fabricated by wet etching was indicated in Fig. 4. Because of high density of pit patterns, which limited reactant gas diffusion, wet chemical fluid could not react successfully during the etching process. This sample showed the worst performance of absorption among hybrid structures.

Figure 4(a) illustrates nanostructures on micro pits obtained using the wet etching method. In contrast to the tips, nanostructures were not observed on the bottom of the pits, although some silver was deposited on the bottom, as shown in Fig. 4(b). Figure 4(c) shows a high-magnification SEM image, indicating nanostructures on the top surface of the pits. During fabrication, many bubbles were observed and remained in the pit area rather than diffusing away. It is possible that nanostructures cannot be formed on the bottom of the pits because bubbles hinder or slow the chemical reaction since the etching end product is SiF_4_, a type of gas that requires air for diffusion.

**Figure 5:**
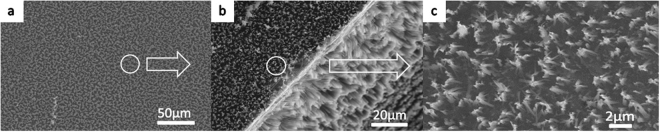
Nanostructures on a flat substrate obtained using the wet etching method: (**a**) top view of a low-magnification micrograph, (**b**) nanostructures on the side wall and surface, and (**c**) high-magnification SEM image of nanostructures on the surfaceof the pits. (**a**) Shows a micrograph of the nanostructures grown on a flat substrate using the wet etching method, captured at low magnification. The growth condition is similar to that of the top surface of the tips and pits with a thick black silicon forest. In addition, (**b**) shows that nanostructures can be fabricated on a vertical side wall with higher grass on the bottom of the side wall. A high-magnification SEM image of the nanostructures on the surface, as presented in (**c**), shows denser grass than that observed for the tips and pits.

Figure 5(a) shows a micrograph of the nanostructures grown on a flat substrate using the wet etching method, captured at low magnification. The growth condition is similar to that of the top surface of the tips and pits with a thick black silicon forest. In addition, Fig. 5(b) shows that nanostructures can be fabricated on a vertical side wall with higher grass on the bottom of the side wall. A high-magnification SEM image of the nanostructures on the surface, as presented in Fig. 5(c), shows denser grass than that observed for the tips and pits.

**Figure 6:**
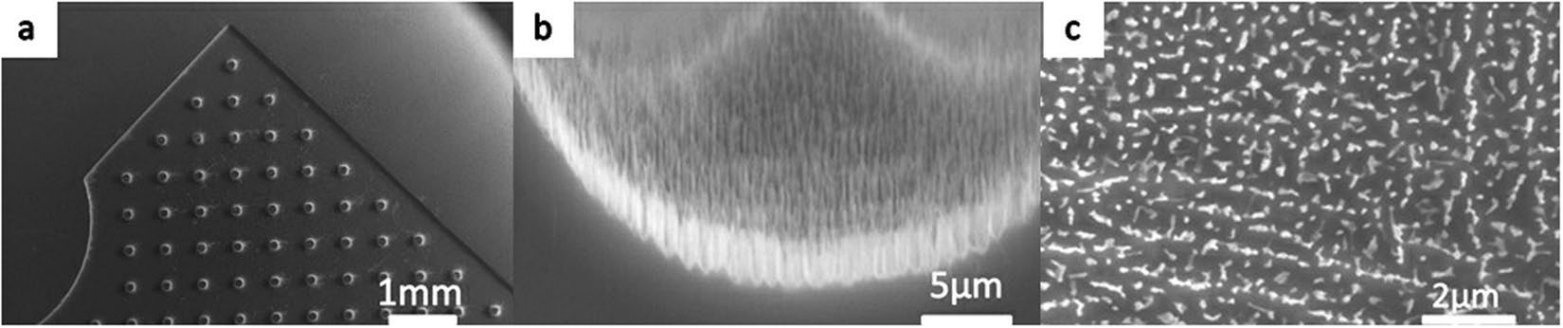
Nanostructures on micro tips obtained using the dry etching method: (**a**) top view of a low-magnification micrograph, (**b**) side view of nanostructures on the profile of the tips, and (**c**) high-magnification SEM image of nanostructures on the profile. (**a**) Shows an array image of micro tips obtained using the dry etching method, with a magnified view in (**b**). (**b**) Indicates that nanostructures are growing well on the profile of the tips, with a height of approximately 1 μm. (**c**) Shows highly dense grass that is even denser than that observed for the flat growth situation using the wet etching method (Fig. 3(c)).

Figure 6(a) shows an array image of micro tips obtained using the dry etching method, with a magnified view in Fig. 6(b). Figure 6(b) indicates that nanostructures are growing well on the profile of the tips, with a height of approximately 1 μm. Figure 6(c) shows highly dense grass that is even denser than that observed for the flat growth situation using the wet etching method (Fig. 3(c)).

**Figure 7:**
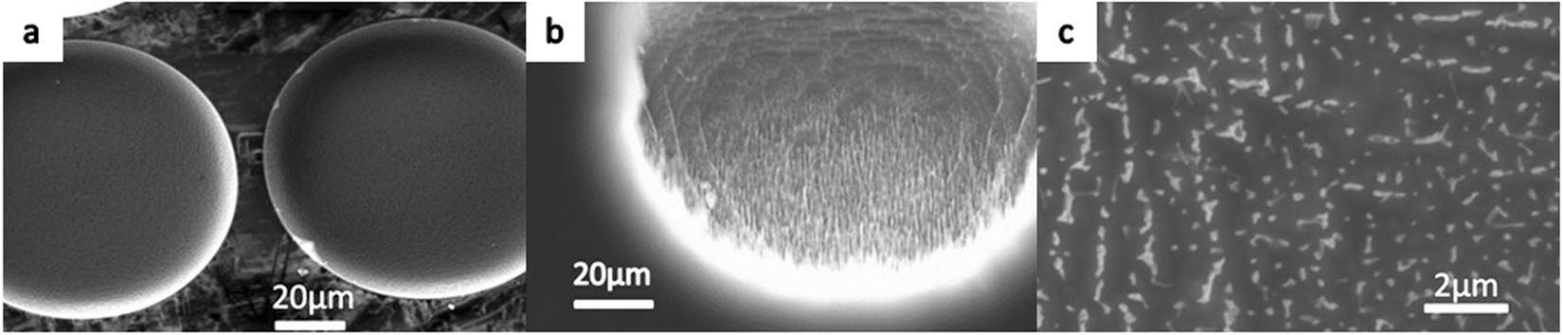
Nanostructures on micro pits obtained using the dry etching method: (**a**) top view of a low-magnification micrograph, (**b**) nanostructures on the profile of the pits, and (**c**) high-magnification SEM image of nanostructures on the profile. (**a**) Shows an image of the nanostructures grown on micro pits using the dry etching method, with a magnified view in (**b**). (**b**) Indicates that nanostructures are growing well on the bottom of the pits, with a height of approximately 1 μm, but no growth occurs on the profile close to the top surface. (**c**) Shows a relatively dense grass that is denser than that obtained for the flat growth conditions using the wet etching method (Fig. 5(c)) but less dense than that obtained for the tip growth condition with the gas method (Fig. 6(c)).

Figure 7(a) shows an image of the nanostructures grown on micro pits using the dry etching method, with a magnified view in Fig. 7(b). Figure 7(b) indicates that nanostructures are growing well on the bottom of the pits, with a height of approximately 1 μm, but no growth occurs on the profile close to the top surface. Figure 7(c) shows a relatively dense grass that is denser than that obtained for the flat growth conditions using the wet etching method (Fig. 5(c)) but less dense than that obtained for the tip growth condition with the gas method (Fig. 6(c)).

**Figure 8:**
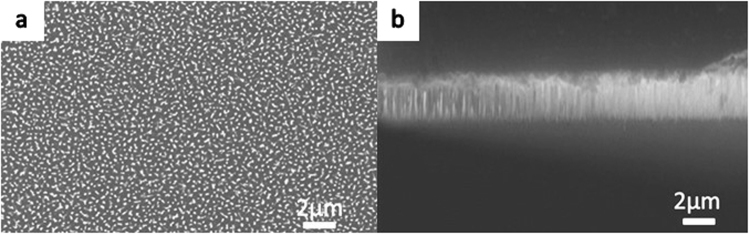
Nanostructures on a flat substrate obtained using the dry etching method: (**a**) high-magnification SEM image of nanostructures on the surface and (**b**) side view of the nanostructures on the surface. Figure 8 shows the top and side view of nanostructures on a flat substrate, obtained using the dry etching method, with a height of approximately 1 μm. This sample has the densest nanostructure among the No. 1~No. 6 samples.

Figure 8 shows the top and side view of nanostructures on a flat substrate, obtained using the dry etching method, with a height of approximately 1 μm. This sample has the densest nanostructure among the No. 1 ~ No. 6 samples.

## Experimental

**Figure 9:**
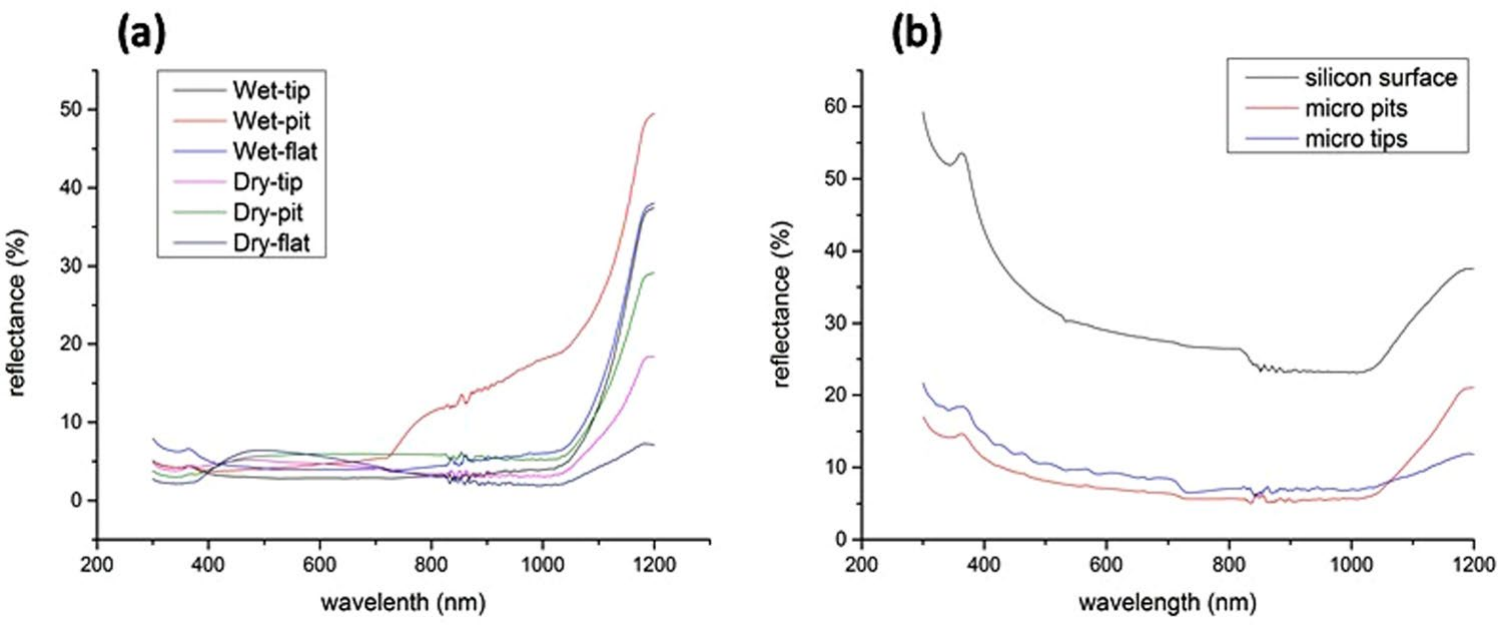
Hemispherical reflectance spectra of black silicon micro-nano hybrid structures obtained for different processes and micro structures. (**a**) Reflectance results of six samples with nanostructures, (**b**) reflectance results of single micro structures and silicon surface with no treatment. As shown in (**a**), six lines are presented, and the reflectivity values of the samples are illustrated. Taking the forbidden energy gap of silicon into consideration, silicon can usually absorb light with wavelengths below 1100 nm. Therefore, wavelengths between 300 nm and 1100 nm are analyzed. As can be inferred from the figure, except for the wet-pit sample (red line) and the dry-flat sample (purple line), the reflectance of the samples decreases slightly with wavelength from 300 nm to 400 nm, remains stable for wavelengths from 400 nm to 1000 nm and increases dramatically for wavelengths from 1000 nm to 1100 nm. The wet-pit sample shows little fluctuation for wavelengths from 300 nm to 700 nm but strongly increases from 700 nm to 1100 nm. The dry-flat sample presents a peak in the wavelength range from 300 nm to 1100 nm, which may originate from the effect of light diffraction. Fluctuations can be seen at wavelengths near 820 nm because the UV-3600 instrument changes light sources from infrared to visible light. Reflectance results of single micro structures and silicon surface with no treatment are shown in (**b**). It can be inferred that silicon with no treatment indicated high reflectance that far surpassed other rough surfaces with single micro structures. The micro pits and tips were only fabricated by ICP without nano structures and the process of them were the same as the No. 1, No. 2, No. 4, and No. 5 samples in first process step.

In this paper, nanostructures were fabricated on micro structures with different profiles: tip, pit and flat surface. Micro structures were dry etched in SF_6_-O_2_ plasma using an ICP (inductive coupled plasma) from the SPTS Company. Black silicon nanostructures were fabricated by two methods, wet etching and dry etching. After the black silicon nanostructures were grown, No. 1 ~ No. 6 samples were observed by a photometer for reflectivity tests. As shown in Fig. 9(a), six lines are presented, and the reflectivity values of the samples are illustrated. Taking the forbidden energy gap of silicon into consideration, silicon can usually absorb light with wavelengths below 1100 nm. Therefore, wavelengths between 300 nm and 1100 nm are analyzed. As can be inferred from the figure, except for the wet-pit sample (red line) and the dry-flat sample (purple line), the reflectance of the samples decreases slightly with wavelength from 300 nm to 400 nm, remains stable for wavelengths from 400 nm to 1000 nm and increases dramatically for wavelengths from 1000 nm to 1100 nm. The wet-pit sample shows little fluctuation for wavelengths from 300 nm to 700 nm but strongly increases from 700 nm to 1100 nm. The dry-flat sample presents a peak in the wavelength range from 300 nm to 1100 nm, which may originate from the effect of light diffraction. Fluctuations can be seen at wavelengths near 820 nm because the UV-3600 instrument changes light sources from infrared to visible light. Reflectance results of single micro structures and silicon surface with no treatment are shown in Fig. 9(b). It can be inferred that silicon with no treatment indicated high reflectance that far surpassed other rough surfaces with single micro structures. The micro pits and tips were only fabricated by ICP without nano structures and the process of them were the same as the No. 1, No. 2, No. 4, and No. 5 samples in first process step. Though the size of micro structures are the same, they performed lower reflectance than those with nano structures and obviously increased in small wavelength range. That is to say, those rough surfaces only shaped with micro structures were less effective than rough surfaces shaped with micro-nano hybrid structures.

**Figure 10:**
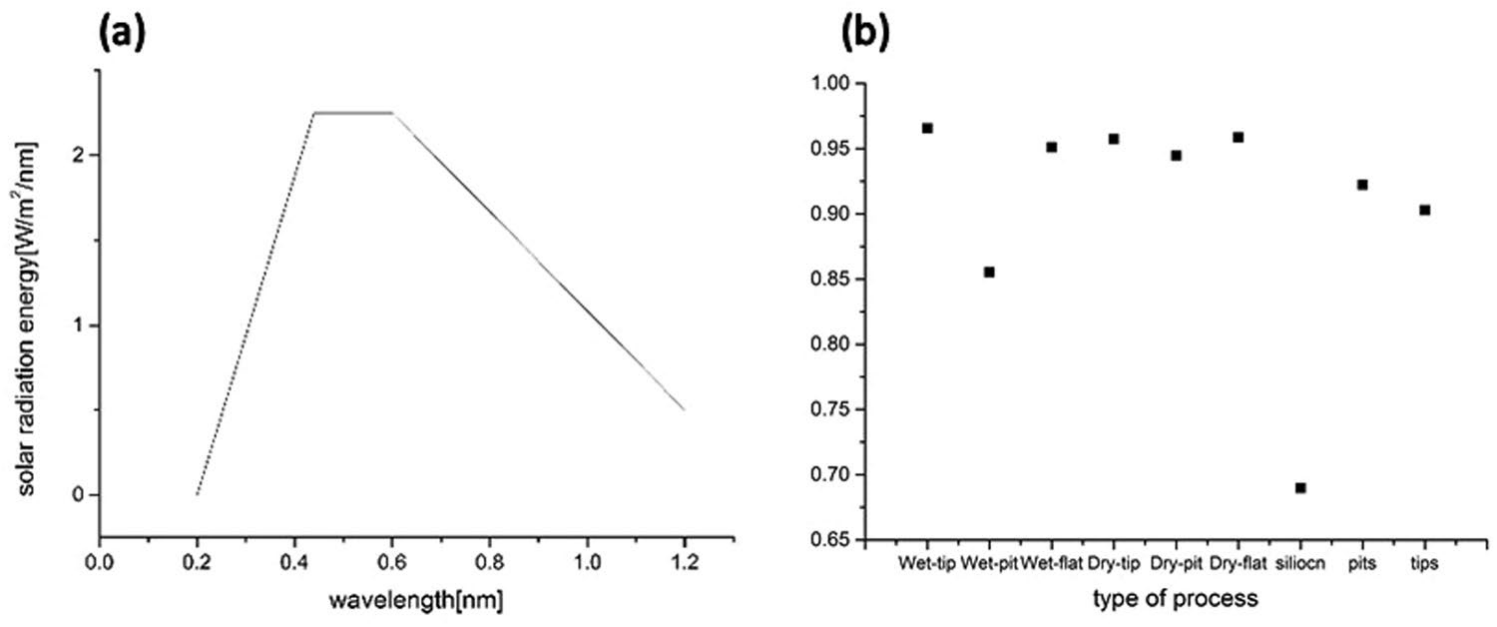
(**a**) Approximate curve of actual solar radiation energy on the earth’s surface from 200 nm to 1200 nm and (**b**) absorption efficiency of micro-nano hybrid structures and micro structures obtained for different processes. Based on the actual solar radiation energy at the earth’s surface^31^, an approximate curve is illustrated in (**a**), which consists of three line segments, to simplify the absorption efficiency of the six micro-nano hybrid black silicon samples and single micro structures samples. As shown in (**b**), the wet-pit samples exhibit the lowest absorption efficiency among No. 1~No. 6 samples, and the wet-tip samples exhibit the highest absorption efficiency among the nine samples. Tips for both wet and dry etching methods show larger absorption efficiencies than pits. The efficiencies of the tip samples exceed the efficiencies of the pit samples by almost 10% for the wet etching method; however, for the dry etching method, the tip and pit samples are close in efficiency. In addition, the tip samples obtained using the wet etching method exhibit a slightly higher efficiency than those obtained using the dry etching method, whereas the flat sample obtained via the wet etching method has a slightly lower efficiency than that of the dry etching method. From the (**b**), silicon surface with no treatment showed lowest among all samples since it has neither micro nor nano structures that contribute to light absorption. The surfaces with pits indicated more 2% efficiency than those with tips since pits may have continuous ring side to capture lights. That is to say, surfaces with pits have more side surface area than those with tips, increasing the probability of reflection. That showed the efficiency of the samples with hybrid structures, single nano structures, single micro structures and no structures, descending from first one to last one.

Based on the actual solar radiation energy at the earth’s surface^72^, an approximate curve is illustrated in Fig. 10(a), which consists of three line segments, to simplify the absorption efficiency of the six micro-nano hybrid black silicon samples and single micro structures samples. The equation of absorption efficiency is given below:

1 $$A={\int }_{300}^{1100}(1-R(\lambda ))\times E(\lambda )\,d\lambda $$

where *A* is the absorption efficiency; *R(λ)* is the reflectance of black silicon at wavelength λ; and *E*(*λ*) is the actual solar radiation energy at the earth’s surface for black silicon at wavelength λ. For the range of integration (300–1100), the minimum value is limited by the lowest wavelength value of the photometer, and the maximum value is limited by the forbidden gap of silicon.

As shown in Fig. 10(b), the wet-pit samples exhibit the lowest absorption efficiency among No. 1 ~ No.6 samples, and the wet-tip samples exhibit the highest absorption efficiency among the nine samples. Tips for both wet and dry etching methods show larger absorption efficiencies than pits. The efficiencies of the tip samples exceed the efficiencies of the pit samples by almost 10% for the wet etching method; however, for the dry etching method, the tip and pit samples are close in efficiency. In addition, the tip samples obtained using the wet etching method exhibit a slightly higher efficiency than those obtained using the dry etching method, whereas the flat sample obtained via the wet etching method has a slightly lower efficiency than that of the dry etching method. From the Fig. 10(b), silicon surface with no treatment showed lowest among all samples since it has neither micro nor nano structures that contribute to light absorption. The surfaces with pits indicated more 2% efficiency than those with tips since pits may have continuous ring side to capture lights. That is to say, surfaces with pits have more side surface area than those with tips, increasing the probability of reflection. That showed the efficiency of the samples with hybrid structures, single nano structures, single micro structures and no structures, descending from first one to last one.

Figure 10(b) also illustrates a relationship among the different micro-nano hybrid structures. The absorption efficiency of the micro-nano hybrid structure obtained using tips is higher than of pits for both the wet and dry etching methods because the nanostructures on the tips are more dense and the nanostructures realize full coverage on the tips but only partial coverage on the pits. In addition, the absorption efficiency of the micro-nano hybrid structure samples using tips with the wet etching method are approximately 2% higher than those using pits with the dry etching method because the tips produced by the wet etching method grew with a higher height of grass than the tips produced by the dry etching method. Higher grass indicates that light trapping is improved. The absorption efficiencies of the micro-nano hybrid structure samples with tips are higher than those without microstructures (flat) for the wet etching method but are similar for the dry etching method. It can be concluded that the tips display a larger area of absorption for solar energy and that the microstructure can absorb secondary light reflections from the other side of the profile.

## Results and Discussions

The wet-tip sample (No. 1) indicated the better performance, high absorption and low reflectance, than other samples. The wet-tip sample was fabricated by dry gas etching that firstly formed micro tips and secondly formed nanograsses on whole profile of tips. On the one hand, nanograsses fabricated by wet metal-assisted etching were higher than using dry etching. On the another hand, it is because nanograsses covered whole profile of tips along with level surface on silicon wafer that realized highest absorption. Light would reflect from one side wall of micro tips to another side wall, and it is a secondary reflection that may increase the probability of being trapped into black silicon. Because all side wall of tips was covered with nanograsses, the light would be absorbed twice or even more times. That is to say, lights that were reflected first time would be re-utilized by black silicon on side wall in second time or even more times. Suppose that the absorption efficiency of bottom side is the same as the curved side (profile), the whole absorption efficiency can be calculated by equation (2). Suppose that the absorption efficiency of bottom side is not the same as the curved side (profile) but attenuating with constant parameter “a” after every reflection, the whole absorption efficiency can be calculated by equation (3).

2 $${E}_{whole}={\int }_{300}^{1100}{\sum }_{i=1}^{n}A{(\lambda )}^{i}\times E(\lambda )\,d\lambda $$

3 $${E}_{whole}={\int }_{300}^{1100}{\sum }_{i=1}^{n}(A{(\lambda )}^{i}\cdot {a}^{i-1})\times E(\lambda )\,d\lambda $$

where *E*_*whole*_ is the whole absorption efficiency of hybrid structures; A(λ) is the absorption efficiency of black silicon at wavelength λ; and E(λ) is the actual solar radiation energy at the earth’s surface for black silicon at wavelength λ. i is the number of times that light really reflect in side wall; a is the attenuating parameter, supposing attenuating remain constant after every reflection. For the range of integration (300–1100), the minimum value is limited by the lowest wavelength value of the photometer, and the maximum value is limited by the forbidden gap of silicon.

**Figure 11:**
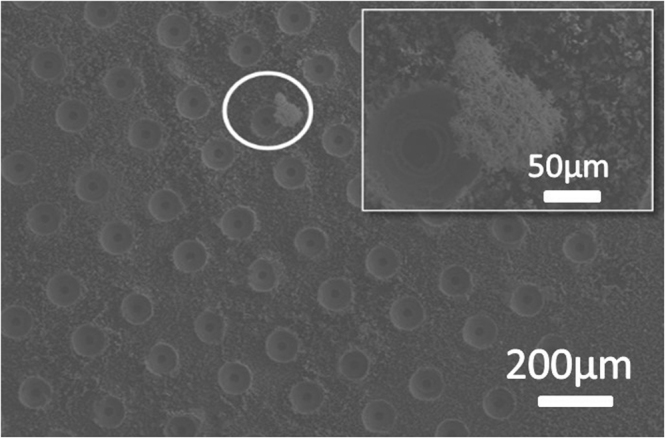
Micrograph of the wet-pit sample and a defect (silver) on the surface. Because the No.1~No.4 samples had different micro-nano hybrid structures, the reflectance showed different variation trends and the absorption had different values. The wet-pit line has a higher reflectance than the other five lines over the wavelength range from 700 nm to 1200 nm because the nanostructures on the pits did not grow well on the profile of the pits, as shown in Fig. 11. Nanostructures cannot be seen in the holes but can be seen at the surface, and the partial silicon (holes) can reflect more light energy than the rest of the silicon (top surface). Figure 11 also indicates a defect on the surface because the silver particles found there were not removed by the HNO_3_ solution. Silver particles hardly existed in tips but few in pits.

Because the No. 1 ~ No. 4 samples had different micro-nano hybrid structures, the reflectance showed different variation trends and the absorption had different values. The wet-pit line has a higher reflectance than the other five lines over the wavelength range from 700 nm to 1200 nm because the nanostructures on the pits did not grow well on the profile of the pits, as shown in Fig. 11. Nanostructures cannot be seen in the holes but can be seen at the surface, and the partial silicon (holes) can reflect more light energy than the rest of the silicon (top surface). Figure 11 also indicates a defect on the surface because the silver particles found there were not removed by the HNO_3_ solution. Silver particles hardly existed in tips but few in pits.

**Figure 12:**
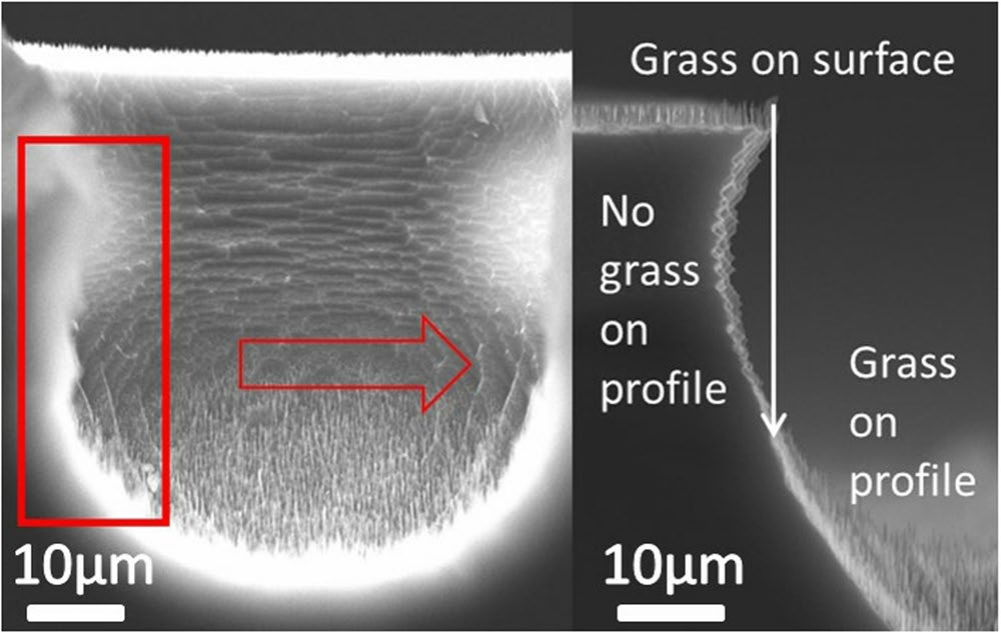
Micrograph of a dry-pit sample and magnification of the side profile. The dry-pit line shows a higher reflectance than the dry-tip results. For the dry etching method, nanostructures can be fabricated on most of the pit profiles; however, nanostructures cannot grow on a small area of the profile, as shown in Fig. 12. Because the plasma is accelerated by the ICP bias power, with a high speed in the vertical direction and a low speed in the horizontal direction, the plasma cannot reach the region that is shielded under the mask or the top silicon surface. In the magnified side profile, the white arrow shows the boundary of the grass growth. Grass can grow on the right side of the arrow but cannot grow on the left side of the arrow. Therefore, the absorption efficiency of a dry-pit sample is lower than that of a sample with tips that grow well over all of the side profile, as shown in Fig. 6(b).

The dry-pit line shows a higher reflectance than the dry-tip results. For the dry etching method, nanostructures can be fabricated on most of the pit profiles; however, nanostructures cannot grow on a small area of the profile, as shown in Fig. 12. Because the plasma is accelerated by the ICP bias power, with a high speed in the vertical direction and a low speed in the horizontal direction, the plasma cannot reach the region that is shielded under the mask or the top silicon surface. In the magnified side profile, the white arrow shows the boundary of the grass growth. Grass can grow on the right side of the arrow but cannot grow on the left side of the arrow. Therefore, the absorption efficiency of a dry-pit sample is lower than that of a sample with tips that grow well over all of the side profile, as shown in Fig. 6(b).

Apart from two fabrication defects in the dry-pit and the wet-pit samples, other nanostructures on the microstructures grew well. However, the pits might show better performance than the tips in theory because light cannot easily escape pits and still achieve full coverage. Therefore, solving these problems in the fabrication of pits is a promising research direction. Moreover, solar radiation in the infrared light range can increase the temperature of a solar cell, which may enhance the solar cell efficiency due to a forbidden gap value decrease. We consider this work as the next step in reducing the reflectance and absorption of solar cells.

## Conclusion

This paper studied the fabrication of micro-nano hybrid structures, measured the reflectances of these structures, and analyzed their absorption based on the integral of reflectivity and solar emissivity at the earth’s surface. The absorption efficiency of the wet-pit sample was found to exhibit the lowest absorption efficiency and that of the wet-tip sample was found to exhibit the highest absorption efficiency among the nine samples. The tips obtained from both the wet and dry etching methods showed higher absorption efficiencies than the pits. The efficiencies of the tips exceed the efficiencies of the pits by almost 10% for the wet etching method; however, for the dry etching method, the tips and pits are similar in efficiency. In addition, the absorption efficiency of the micro-nano hybrid structure with tips fabricated using the wet etching method is approximately 2% higher than that with pits fabricated with the dry etching method. The absorption efficiency of the micro-nano hybrid structure with tips is higher than that without a microstructure (flat) for the wet etching method, but the two values are similar for the dry etching method. According to results and comparisons between surfaces with single micro structures and those with hybrid structures, the former ones were less effective than latter ones. To sum up, silicon wafers with hybrid structures have higher absorption than those with single nano structures. Besides, silicon wafers with with single nano structures have higher absorption than those with single micro structures. Finally, silicon wafers with with single micro structures have higher absorption than those with no treatment. Among hybrid structures, tips with wet treatment in second step showed higher performance than tips and pits with dry treatment in second step, and pits with wet treatment in second step showed worst performance.
